# Temporomandibular disorders in women with psoriatic arthritis by history of psoriasis: a cross-sectional study

**DOI:** 10.1007/s00296-026-06318-7

**Published:** 2026-10-14

**Authors:** Negin Yekkalam, Anders Wänman, Göran Isacsson, Aylin Canbek, Milad Rizk

**Affiliations:** 1 Department of Odontology, Orofacial Pain and Jaw Function, Umeå University, Umeå, Sweden; 2 Department of Orofacial Pain and Jaw Function, Västmanland Hospital Västerås, Västerås, Sweden; 3 Department of Rheumatology, University Hospital Zurich, Zurich, Switzerland; 4 Hemdal Primary Care Center, Region Västmanland, Västerås, Sweden

**Keywords:** Temporomandibular joint disorders, Psoriatic arthritis, Cross-sectional studies, Patient reported outcome measures, Treatment groups

## Abstract

Temporomandibular joint involvement in psoriatic arthritis may result in impaired jaw function and reduced oral health-related quality of life. Patients with PsA have generally been analyzed as a single group regardless of psoriasis history, despite a reported association between psoriasis and TMJ involvement. To assess TMD symptoms, patient-reported outcome measures, and self-reported beneficial effects of systemic therapies and TMD-related treatment modalities in women with psoriatic arthritis, stratified by psoriasis history. A digital questionnaire was sent to members aged ≥ 18 years of the Swedish Rheumatism Association from March through September 2022. The study included 27 women with self-reported PsA diagnosed within the healthcare system. Descriptive analyses were performed to describe socio-demographic, disease-related, TMD-related, and patient-reported outcome measures stratified by psoriasis history. Pain during jaw activity (77.8%), TMJ sounds (74.1%), and morning jaw stiffness/pain/fatigue (66.6%) were the most reported symptoms in the total sample. The proportions were higher among women with a history of psoriasis compared with those without a history of psoriasis (81.8% vs. 75.0%, 81.8% vs. 68.8%, and 72.7% vs. 62.5%, respectively). Those with psoriasis history had higher median (Q1–Q3) JFLS-8 scores [18 (5–22) vs. 11.5 (8–17.5)] and higher mean (SD) OHIP-14 scores (16.7 ± 7.7 vs. 9.6 ± 7.4) than those without psoriasis history. A perceived beneficial effect of systemic PsA medication on TMD symptoms was reported by 40% and 30%, respectively. Occlusal adjustment in the psoriasis-history group and bite splint therapy in the other group were reported as the most received and most beneficial treatments in the respective. Women with PsA reported TMD symptoms and impaired patient-reported outcomes, with higher proportions reported in the psoriasis-history group. Self-reported beneficial effects of systemic and TMD-related treatments varied between groups. Findings should be interpreted cautiously given the small sample size and descriptive design. Further studies with larger samples are recommended.

**Supplementary Information:**

The online version contains supplementary material available at https://doi.org/10.1007/s00296-026-06318-7.

## Introduction

Psoriatic arthritis (PsA) is a heterogeneous inflammatory disease with both intra- and extra-articular manifestations. Musculoskeletal manifestations include peripheral arthritis affecting the hands, feet, knees, and ankles, as well as axial disease and dactylitis. Extra-articular manifestations may include mucosal involvement and other systemic sites [1]. PsA is also associated with fibromyalgia, mood disorders, obesity, osteoporosis, fatigue, and sleep disturbances, all of which contribute significantly to reduced health-related quality of life [2, 3].

In the adult general population, PsA prevalence is approximately 0.1–0.2% [2], while in Sweden it is about 0.35% [4]. The age of onset is commonly between 30 and 50 years [5], and late onset is associated with a higher burden of comorbidities and peripheral polyarthritis [6]. Currently, there is no biomarker for PsA, making diagnosis challenging [7]. This is particularly problematic when PsA overlaps with other rheumatic diseases, as it may lead to irreversible joint damage and functional disability [8].

PsA most commonly develops in individuals with pre-existing psoriasis, affecting about 20–30% of patients after an average latency of 10 years [9]. Psoriasis is a chronic inflammatory skin disease that can involve the nails, scalp, and mucous membranes, with an approximate prevalence of 0.1–1% in the general population [10]. Early and effective treatment of psoriasis may reduce the risk of PsA development. The mechanisms of transition from psoriasis to PsA are unclear, however severe skin involvement [11], elevated inflammatory markers, and a smoking history are associated with increased risk of PsA development [9]. Importantly, a known history of psoriasis may facilitate earlier diagnosis of PsA [10].

The pathogenesis of PsA results from a complex interaction between polygenic (HLA and non-HLA genes) and environmental factors [12], which may activate inflammatory pathways leading to arthropathy and erosive bone changes [13]. Thus, targeting tumor necrosis factor-alpha (TNF-α), interleukin (IL)−17, and IL-23, particularly with disease-modifying antirheumatic drugs (DMARDs) are central in treatment to reduce pain, alongside multidisciplinary rehabilitation and physical activity [14].

Temporomandibular joint (TMJ) involvement can also occur in PsA, potentially resulting in temporomandibular disorders (TMD), an umbrella term for a group of conditions affecting the TMJ, masticatory muscles, and associated structures [15]. The prevalence of TMD in PsA varies considerably across studies, depending on the diagnostic methods and study population. Nevertheless, available evidence suggests that TMD signs and symptoms are more common in patients with PsA than in healthy controls [16–18]. Women appear to be more affected, possibly due to hormonal, genetic, sociocultural, and behavioral factors [19].

Clinical manifestations of TMD include myalgia, arthralgia, limited range of jaw movement, swelling, joint sounds, and jaw locking [20]. Consequently, it may significantly impair jaw function and oral health-related quality of life [19]. TMD can also be asymptomatic despite the presence of TMJ changes on radiographic imaging [18]. Since TMD symptoms and imaging findings are not specific to PsA [21, 22], TMD may be either part of PsA or a coexisting condition. This complicates diagnosis and management in this patient population, where a combination of systemic pharmacological and TMD-specific interventions may be important to reduce pain and improve jaw function [20].

Overall, studies investigating TMJ involvement in patients with PsA are scarce compared to other rheumatic diseases [16, 21, 22]. Moreover, patients with PsA have generally been analyzed as a single group regardless of psoriasis history [22, 23], despite previous reports suggesting an association between psoriasis and TMJ involvement [24]. In addition, among the available TMD treatment approaches, most studies focused on TMJ intra-articular cortisone injection in patients with PsA [25]. The first aim of this study was to assess the presence of TMD symptoms and patient-reported outcomes in individuals with PsA, stratified by psoriasis history. The second aim was to evaluate the self-reported beneficial effects of systemic therapies and TMD-related treatment modalities in individuals with PsA, also stratified by psoriasis history.

## Materials & methods

### Study design and participants

This study was designed as a questionnaire-based cross-sectional study. Participants were members in the Swedish Rheumatism Association (Reumatikerförbundet). Inclusion criteria were individuals over 18 years of age with one or more confirmed diagnoses of systemic autoimmune rheumatic diseases within the healthcare system. Ethical approval was granted by Etikprövningsmyndigheten (Swedish Ethical Review Authority) on 2 March 2022 (Dnr 2022-00809-01). Prior to participation, all participants confirmed that they had received sufficient information about the study and provided informed consent to participate by selecting “Yes” on the study platform. Data were collected between March and September 2022.

The study was conducted in accordance with the ethical principles of the World Medical Association’s Declaration of Helsinki. The STROBE guidelines for observational studies were followed [26].

**Fig. 1:**
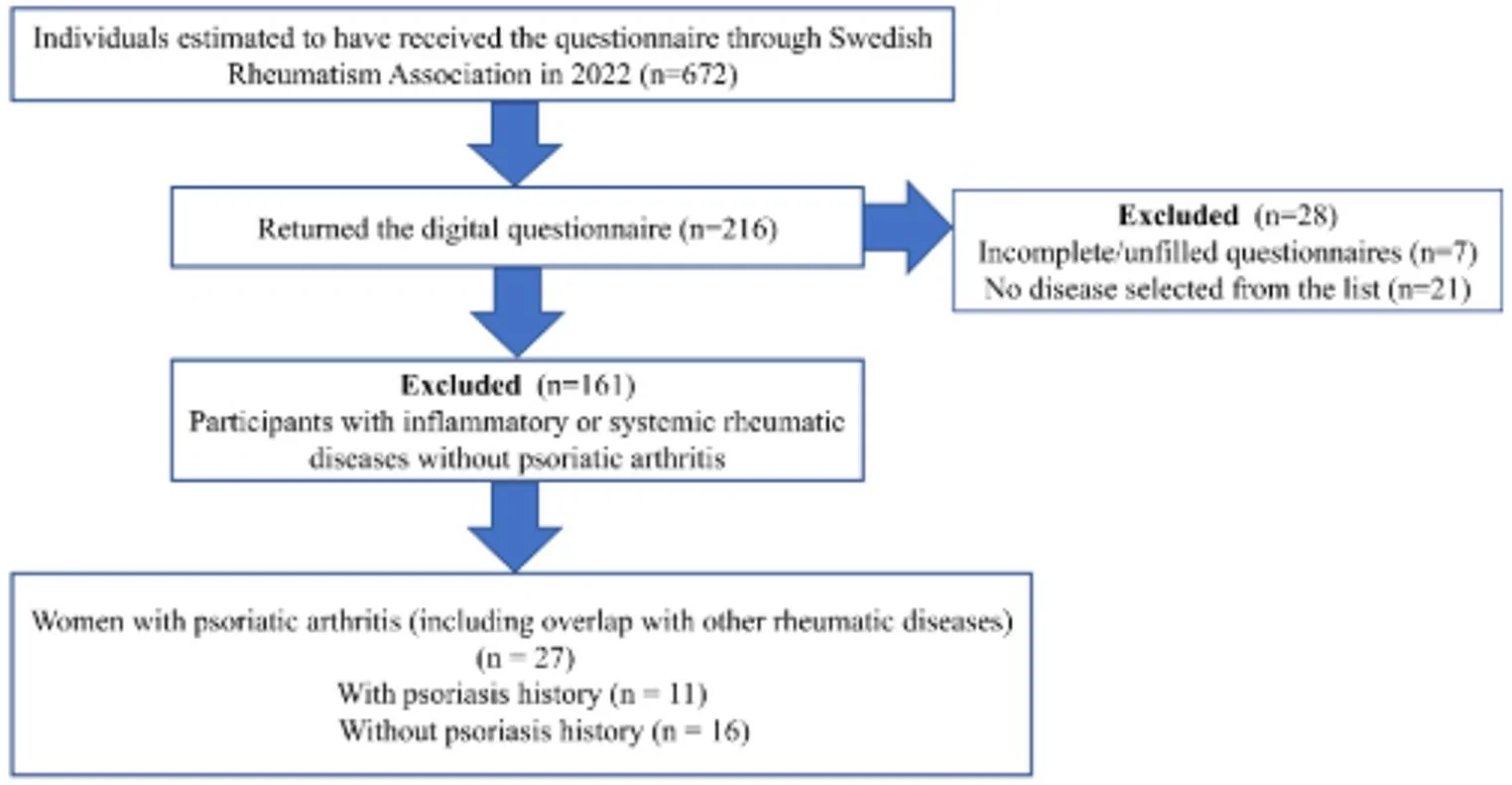
Inclusion and exclusion of participants in this study. Overlap refers to coexisting reported diagnoses of other inflammatory rheumatic diseases such as rheumatoid arthritis, ankylosing spondylitis, and inflammatory bowel disease–related arthritis

The sample size was determined by the number of eligible individuals who responded and consented to participate. No formal sample size calculation was performed due to the exploratory nature of the study. The final cohort consisted of 27 women with self-reported psoriatic arthritis. Inclusion and exclusion criteria are shown in Fig. 1.

### Questionnaire

Data were collected using a single self-administered questionnaire in the Swedish language consisting of 57 questions. The questionnaire was distributed via a digital link on the Facebook page of the Swedish Rheumatism Disease Association members. Two reminders were posted on both Facebook and the member website to reduce the selection and non-response bias. Only women responded to the questionnaire in the present study.

The study measures included socio-demographic, health-related, disease-related, and TMD-related factors.

### Socio-demographic factors

Age was registered in years. Country of birth was classified as either Sweden or other. In this study, the “other” category included women born in Finland and Norway. Self-reported living conditions were categorized as either living alone or with others. Employment status was dichotomized as working versus not actively working (e.g., unemployed, on sick leave, parental leave, retired, or on other forms of leave).

### Health-related factors

Self-reported general and oral health were assessed on a 5-point scale and dichotomized into good (very good/good) and poor (fair/poor/very poor). Self-reported fatigue was assessed using a yes/no question with no specified time frame. Widespread pain was assessed by recording pain in 19 body sites and joints during the past 7 days to calculate the Widespread Pain Index. Widespread pain was defined as pain in seven or more sites (WPI ≥ 7) [27]. Two additional yes/no questions assessed the presence of clinically diagnosed fibromyalgia and hypermobility.

### PsA-related factors

Age of PsA diagnosis (years), pattern of PsA (PsA only or overlap with other rheumatic diseases), family history of PsA, psoriasis history and latency period (years), nail involvement, and Body Mass Index categories were recorded. Smoking status was classified as never, former, or current smoker. Self-reported osteoporosis and medication-based indicator for depression (“Do you take medication for depression?”) were assessed with yes/no options.

Self-reported beneficial effect of medication for PsA after 3–6 months use was assessed, including different types of disease-modifying antirheumatic drugs (DMARDs), non-steroidal anti-inflammatory drugs (NSAIDs), corticosteroids, and analgesics. Participants could report all medications used by name. In the analysis, DMARDs were classified as monotherapy (1 DMARD) or combination therapy (2 DMARDs). Medication classification is shown in Supplementary information Table 1.

Perceived adequate PsA-related knowledge among dental staff was assessed by asking following question: “At your most recent dental visit, did you feel that the dental care staff had sufficient knowledge about your illness to provide you with good dental care? " (yes/no).

### TMD-related factors

The 3Q/TMD, a validated three-question screening tool for TMD, was used to identify possible TMD. Q1 assessed pain in the temple, face, TMJ, or jaw; Q2 assessed pain during mouth opening or chewing; and Q3 assessed jaw locking or locking episodes, all occurring at least once per week. At least one positive response was classified as a positive screening result [28]. 

TMD symptoms were assessed using six yes/no questions derived from the TMD Pain Screener and the DC/TMD Symptom Questionnaire [29], covering jaw pain, stiffness or fatigue, pain during jaw function, joint noises, jaw luxation/dislocation, and jaw locking. Participants reporting any of these symptoms were classified as having TMD symptoms.

Self-reported bruxism was assessed by asking whether participants clenched or ground their teeth. A positive response was classified as bruxism. *Self-reported bite stability* was assessed by asking about the participants’ perception of how the teeth fit together (stable vs. unstable; yes/no options).

Perceived knowledge of TMD in healthcare was assessed by asking participants whether jaw symptoms had been addressed at the most recent healthcare visit with yes/no response options.

### Patient-reported outcome measures

Jaw functional limitation was assessed using the 8-item Jaw Functional Limitation Scale (JFLS-8), which evaluates limitations in mastication, vertical jaw mobility, and verbal and emotional expression over the past 30 days. Items are rated on an 11-point scale (0–10), with total scores ranging from 0 to 80; higher scores indicate greater functional limitation [30]. 

Oral health-related quality of life (OHRQoL) was assessed using the validated 14-item Oral Health Impact Profile (OHIP-14) over the past 30 days. The questionnaire assesses oral function, orofacial pain, orofacial appearance, and psychosocial impact. Items are rated on a 5-point scale (0–4), with total scores ranging from 0 to 56; higher scores indicate greater impairment [31]. 

Received TMD-related treatment was captured through self-reported treatment options based on the Swedish national guidelines [32], including no treatment need, counselling to avoid jaw clenching behavior, advice on jaw exercises, occlusal adjustments, bite splint therapy, anti-inflammatory medication, TMJ intra-articular cortisone injection, anesthetic or botulinum toxin injections in the jaw muscles, orthodontic treatment, arthrocentesis, and jaw surgery such as discectomy, and prosthetic TMJ replacement.

Perceived beneficial effects of received TMD-related treatment was assessed using a question asking participants which treatments they perceived as beneficial.

**Fig. 2:**
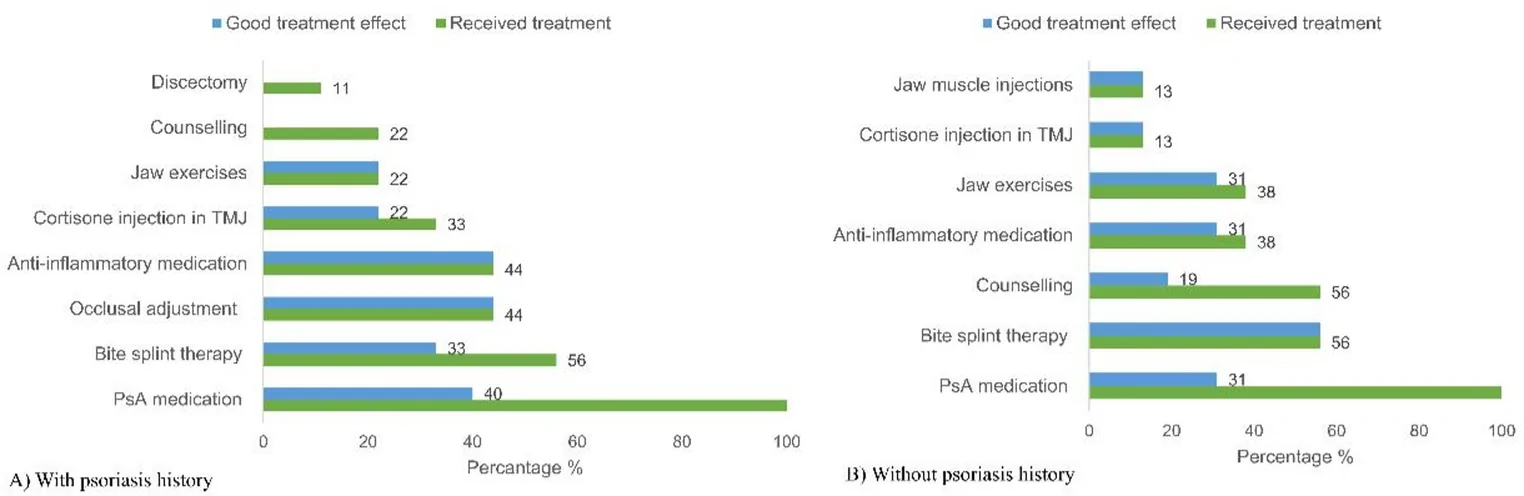
Reported TMD treatment modalities and their perceived beneficial effects in women with PsA: (**a**) with psoriasis history (*n* = 10); (**b**) without psoriasis history (*n* = 16). Jaw muscle injections refer to botulinum toxin or local anesthetic injections

Self-reported effect of PsA medication on TMD was assessed among participants reporting a beneficial effect of their medication for PsA, to determine whether this effect was also reflected in TMD symptoms. This was evaluated by asking whether participants had noticed any changes in jaw-related problems during 3–6 months of medication use. Response options included: no change, improved, worsened, or uncertain. Only improved responses are shown on Fig. 2.

### Statistical analysis

Data analysis was performed using Stata version 14. The total sample (*n* = 27) was described in terms of socio-demographic characteristics, health-related, disease-related, and TMD-related factors stratified by psoriasis history, using absolute numbers and proportions. No statistical comparisons were done due to the small sample size. Patient-reported outcome measures were assessed among participants with TMD symptoms (*n* = 26/27). JFLS-8 scores were presented median (first quartile–third quartile [Q1–Q3]), and OHIP-14 as mean ± (standard deviation, SD). JFLS-8 item data are presented in Supplementary Information Table 2, and OHIP-14 items, total score, and subdomain data in Supplementary Information Table 3.

Self-reported perceived beneficial effects of systemic PsA medication on TMD, as well as received TMD-related treatments and their perceived benefits, were described according to psoriasis history. In the PsA group with a history of psoriasis (*n* = 11), 10 individuals reported TMD symptoms, of whom 9 received TMD-related treatment; therefore, analyses were based on these 10 individuals. In the group without a history of psoriasis (*n* = 16), all individuals reported TMD and received treatment.

Missing data were described in footnotes under each table, and the proportions and absolute numbers were presented for the available data. Missing data included one for country of birth, one for working status (Table 1), and three for self-reported osteoporosis (Table 2).

## Results

**Table 1:** Socio-demographic and self-reported health characteristics of women with psoriatic arthritis (PsA) by history of psoriasis

Variable	With psoriasis history (*n* = 11) *n* (%)	Without psoriasis history (*n* = 16) *n* (%)	Total (*n* = 27) *n* (%)
**Age** (years), mean ± SD	55.3 ± 2.8	54.8 ± 1.9	55 ± 8.2
**Country of birth** ^**1**^			
Sweden	9 (81.8)	15 (93.8)	24 (88.9)
Other (Finland and Norway)	2 (18.2)	0	2 (7.4)
**Living with others**	4 (36.4)	8 (50)	12 (44.4)
**Working** ^**2**^	4 (36.3)	9 (60)	13 (50)
**Self-reported health-related factors**			
Poor oral health	3 (27.3)	8 (50)	11 (40.7)
Poor general health	9 (81.8)	12 (75)	21 (77.8)
Fatigue	11(100)	16 (100)	27 (100)
Widespread Pain Index ≥ 7	7 (63.6)	6 (37.5)	13 (48.1)
Clinically diagnosed fibromyalgia	4 (36.4)	4 (25)	8 (29.6)
Clinically diagnosed hypermobility	7 (63.6)	6 (37.5)	13 (48.1)

*SD *standard deviation. ^1^One missing value for country of birth in women with PsA without a history of psoriasis. ^2^One missing value for working status in both groups. Definitions of the reference groups are provided in the Method section.

The mean age was similar in both groups (55.3 vs. 54.8 years), and the majority were born in Sweden. Being actively working was more commonly reported in the group without psoriasis history. In the total sample, poor general health and fatigue were reported by 77.8% and 100%, respectively. In the group with psoriasis history, the proportions with a Widespread Pain Index ≥ 7, fibromyalgia and hypermobility were higher compared with PsA without psoriasis history.

**Table 2:** Self-reported disease-related and treatment characteristics in women with psoriatic arthritis (PsA), with psoriasis history (*n* = 11), and without psoriasis history (*n* = 16)

Variable	With psoriasis history *n* (%)	Without psoriasis history *n* (%)	Total *n* (%)
**PsA disease pattern** ^1^			
PsA only	9 (81.8)	12 (75)	21 (77.8)
PsA overlap with other inflammatory diseases	2 (18.2)	4 (25)	6 (22.2)
**Age at PsA diagnosis**, years mean (range)	32.4 (10–48)	39.4 (10–60)	36.6 (10–60)
**Family history of PsA**	8 (72.7)	13 (81.3)	21 (77.8)
**Latency period**^**2**^, years (mean ± SD)	9.5 ± 7.4	NA	
**Nail involvement**	8 (72.7)	NA	
**Body Mass Index (BMI**)			
Normal weight (18.5–24.9)	0	1 (6.2)	1 (3.7)
Overweight (25.0–29.9)	5 (45.5)	11 (68.8)	16 (59.3)
Obese (≥ 30.0)	6 (54.5)	4 (25)	10 (37)
**Smoking history**			
Never smoker	7 (63.6)	13 (81.2)	20 (74.1)
Former smoker	4 (36.4)	3 (18.8)	7 (25.9)
**Comorbidities**			
Self-reported osteoporosis^**3**^	4 (40)	4 (28.6)	8 (29.6)
Medication-based indicator of depressive symptoms	4 (36.4)	4 (25)	8 (29.6)
**Self-reported beneficial effect of medication for PsA (3–6 months use)**			
1 DMARD (monotherapy)	7 (63.6)	8 (50)	15 (55.6)
2 DMARDs (combination therapy)	1 (9.1)	3 (18.8)	4 (14.8)
Corticosteroid (Cortisone, Prednisolone)	2 (18.2)	4 (25)	6 (22.2)
NSAIDs	5 (45.5)	7 (43.8)	12 (44.4)
Analgesics	4 (36.4)	2 (12.5)	6 (22.2)
**Perceived adequate PsA-related knowledge among dental staff**	7 (63.6)	9 (56.3)	16 (59.3)

^1^PsA overlap with other inflammatory diseases include Rheumatoid Arthritis, Ankylosing Spondylitis, and inflammatory bowel disease–related arthritis, **NA** indicates not applicable. ^2^ Latency period is the years between the psoriasis diagnosis and the PsA diagnosis. ^3^ Three missing values for self-reported osteoporosis. *DMARDs* disease-modifying antirheumatic drugs, *NSAIDs* non-steroidal anti-inflammatory drugs

In the total sample, the majority had a diagnosis of PsA only, a family history of PsA, and were overweight. The mean age at PsA diagnosis was higher in the group without psoriasis history. The mean latency period was 9.5 years with large individual variation. The proportions of former smokers, self-reported osteoporosis, and medication-based depressive symptoms were higher in the group with a history of psoriasis. DMARD (monotherapy) and NSAIDs were the most reported medications with a beneficial effect on PsA in the sample. Overall, more than half of participants perceived adequate knowledge about PsA among dental staff.

**Table 3:** TMD characteristics in women with psoriatic arthritis (PsA), and patient-reported outcome measures in women with PsA and reported TMD symptoms

Variable	With psoriasis history *n* (%)	Without psoriasis history *n* (%)	Total *n* (%)
**Screening 3Q/TMD**	9 (81.8)	14 (87.5)	23 (85.2)
Q1: pain in the temple, face, TMJ or jaw ≥ 1/week	8 (72.7)	12 (75)	20 (74.1)
Q2: pain in the temple/face/jaw during jaw functions ≥ 1/week	9 (81.8)	8 (50)	17 (63)
Q3: Jaw locking ≥ 1/week	1 (9.1)	1 (6.3)	2 (7.4)
**Morning jaw pain/stiffness/fatigue**			
No	3 (27.3)	6 (37.5)	9 (33.3)
Unilateral	3 (27.3)	4 (25)	7 (25.9)
Bilateral	5 (45.4)	6 (37.5)	11 (40.7)
**Pain affected by jaw activity (last 30 days)**	9 (81.8)	12 (75)	21 (77.8)
**TMD dysfunction-related symptoms**			
TMJ sounds (clicking and/or crepitation)	9 (81.8)	11 (68.8)	20 (74.1)
Jaw locking	0	1 (6.3)	1 (3.7)
**Pattern of TMD Symptoms**			
No symptoms	1 (9.1)	0	1 (3.7)
TMD pain only	1 (9.1)	5 (31.2)	6 (22.2)
TM dysfunction only	1 (9.1)	2 (12.5)	3 (11.1)
TMD pain and/or dysfunction	10 (90.9)	16 (100)	26 (96.3)
**Self-reported bruxism**	11 (100)	9 (56.3)	20 (74.1)
**Self-reported bite instability**	4 (36.4)	7 (43.8)	11 (40.7)
**Asked about jaw symptoms at the most recent healthcare visit**	1 (9.1)	0	1 (3.7)
**Patient outcome measures**	(*n* = 10)	(*n* = 16)	(*n* = 26)
**JFLS-8 total score**,** median [Q1–Q3]**	18 (5–22)	11.5 (8 − 17.5)	13.5 (7 − 21)
**OHIP-14 total score**,** mean ± SD**	16.7 ± 7.7	9.6 ± 7.4	12.3 ± 8.2
**OHIP-14 subdomains**			
Oral function	1.0 ± 1.3	0.4 ± 1.0	0.6 ± 1.1
Orofacial pain	3.9 ± 2.5	2.9 ± 2.4	3.3 ± 2.4
Orofacial appearance	3.0 ± 1.9	2.5 ± 1.9	2.7 ± 1.8
Psychosocial impact	2.0 ± 1.2	0.4 ± 0.8	1.0 ± 1.2

*JFLS-8* Jaw Functional Limitation Scale-8, *OHIP-14* 14-item Oral Health Impact Profile, *Q1–Q3* first quartile–third quartile, *SD* standard deviation

Overall, more than 80% reported at least one positive 3Q-TMD symptom. Around 40% reported bilateral morning jaw pain/stiffness/fatigue, and 74.1% TMJ sounds, with a higher proportion among those with psoriasis history. Almost all reported TMD pain and/or dysfunction symptoms. No participants reported jaw luxation, but one had jaw locking. In the total sample, 74.1% reported bruxism and 40.7% bite instability. Only one individual in the entire sample had been asked about jaw symptoms at the most recent healthcare visit. JFLS-8 and OHIP-14 scores were higher in women with psoriasis history. In the total sample, orofacial pain was the most affected OHIP-14 subdomain, followed by orofacial appearance.

In the group with psoriasis history, 40% reported a beneficial effect of PsA medication under 3–6 months use, (50%) no changes, and (10%) were uncertain (Fig. 2). In the group without psoriasis history, corresponding proportions were 31%, 44%, and 25%, respectively. The most commonly received TMD treatments included bite splint therapy, anti-inflammatory medication, occlusal adjustment, TMJ intra-articular cortisone injection, counselling, and jaw exercises. Occlusal adjustment and anti-inflammatory medication were perceived as the most beneficial treatments in the group with a history of psoriasis, while bite splint therapy was perceived as the most beneficial treatment in the group without a history of psoriasis.

## Discussion

To our knowledge, this is the first cross-sectional study in women with psoriatic arthritis (PsA) assessing TMD symptoms stratified by psoriasis history. Almost all women reported TMD symptoms with higher proportions of pain-related symptoms and TMJ sounds in the group with psoriasis history. They also had higher total scores on the JFLS-8 and OHIP-14. A perceived beneficial effect of PsA medication on TMD symptoms after 3–6 months of use was reported by 40% in the psoriasis-history group and 30% in the other group. Occlusal adjustment in the psoriasis-history group and bite splint therapy in the other group were reported as the most received treatments and were perceived as most beneficial. Overall, the findings should be interpreted with caution due to the small sample size and descriptive nature of the study.

Pain during jaw activity (77.8%), TMJ sounds (74.1%), and morning jaw stiffness/pain/fatigue (66.6%), were the most reported symptoms in the total sample. Importantly, the proportions reporting the screening pain-related questions Q1 (74.1%) and Q2 (63.0%) were comparable to those reporting TMD pain-related symptoms. These findings highlight the potential applicability of 3Q/TMD as a quick screening tool in routine healthcare practice, which may facilitate the identification of patients with TMD pain-related symptoms. Notably, all but one of the participants reported not being asked about jaw symptoms at their most recent healthcare visit.

To our knowledge, no studies have stratified TMD in PsA based on psoriasis history, which limits direct comparison with our findings. However, higher TMD symptoms and signs in PsA patients with a psoriasis history compared with healthy controls have been reported [16, 17, 33]. In the study by Croncoli et al., TMD signs and symptoms were reported in 80% of PsA patients (*n* = 25) versus 20% in controls (*n* = 110) [16]. Previous studies have reported associations between TMJ involvement in PsA and disease severity and duration, the number of affected joints, and enthesitis [17, 18]. In our sample, women with psoriasis history were diagnosed at a younger age (32.4 vs. 39.4 years) despite having a similar mean age between the groups. Pre-existing psoriasis and nail involvement have also been reported to be associated with an increased risk and progression of TMJ arthritis [20]. In our study, nail involvement was reported by 72.7% of participants, consistent with findings of Dervis et al. in a larger sample (70%) [17].

Unlike several other rheumatic diseases, TMJ structural changes in PsA are characterized by both degenerative and proliferative patterns [21]. Such structural changes have been identified in 31–41% of patients with PsA (*n* = 110) compared with 12–13% of controls (*n* = 112) using panoramic radiography [21, 22]. In addition to systemic therapy, local treatment such as TMJ intra-articular cortisone injection has been reported to improve symptoms and jaw function with short- and long-term effects remaining unclear [25]. In our study, a beneficial effect of TMJ intra-articular treatment was also reported. With disease progression, TMJ structural changes may lead to crepitation, functional limitation and irreversible occlusal alterations. The reported beneficial effects of occlusal adjustments only in the group with psoriasis history may reflect management of occlusal alterations secondary to TMJ structural changes, although the contribution of disease duration is unclear. Generally, occlusal adjustment is not the first-line modality and should be limited to selected cases [32]. Other modalities including bite splint therapy and anti-inflammatory medication have previously been reported to have a significant influence on TMJ degenerative joint disease improvement [34]. In our study, the underlying diagnosis for the reported beneficial effects of such treatments is unclear, as morning jaw pain/fatigue/stiffness may reflect both arthralgia and myalgia. Both treatments may also benefit myogenous TMD pain [32]. It has been reported that generalized joint hypermobility, with increased ligamentous laxity, may contribute to disc displacement [35]. This may provide one possible explanation for the high proportion of TMJ sounds reported in our sample, independently of systemic disease. Clinically diagnosed hypermobility was reported in 48.1% of women in the current study.

PsA may also contribute to the development of central sensitization and fibromyalgia (FM) [36]. Impaired central descending pain modulation has been associated with a poorer response to six months of TNF-α inhibitor therapy in patients with inflammatory arthritis [37]. FM is substantially more prevalent in patients with PsA than in the general population (10–64% vs. 2–4%) [36]. Both conditions share key symptoms such as pain, stiffness, and fatigue, which can complicate diagnosis and disease activity assessment [36]. Patients with concomitant PsA and FM are more likely to use combination therapies, NSAIDs, and analgesics and are less likely to achieve minimal disease activity [38]. In our study, 29.6% of women reported a clinical diagnosis of FM, and 48% exhibited widespread pain (WPI ≥ 7), with higher proportions in the psoriasis-history group. Widespread pain has also been reported as a factor associated with poor DMARD outcomes [38].

Depression is another factor associated with FM and widespread pain and is a strong negative predictor of disease remission. It may result from chronic pain, functional limitations, treatment burden, loss of income, and effects on family relationships [38]. Depression has also been identified as a significant predictor of TMD onset and persistence [39]. In our study, 29.6% reported antidepressant use, which was used as an indicator of possible depressive symptoms in accordance with previous Swedish research [40].

Other factors, including elevated BMI and smoking have been associated with higher disease activity and poorer DMARD response in PsA [9, 36]. Although the relationship between elevated BMI and TMD remains unclear [41], smoking has been associated with a higher prevalence and severity of TMD [42]. In our study, 96.3% of participants had a BMI ≥ 25, and 25.9% were former smokers.

Overall, these findings suggest that comorbidities in PsA may contribute to self-reported poor general health, which has been associated with TMD [42]. These factors may also be relevant to the reported limited improvement in TMD symptoms despite good effects of systemic treatment on PsA in this study. Thus, a multidisciplinary approach may be warranted. However, TMD may also occur independently of PsA and may require specific management.

### Patient-reported outcome measures

Women with PsA and TMD symptoms had a higher median JFLS-8 score [13.5 (Q1-Q3: 7–21)] than Swedish population norms (close to 0) [43], indicating substantial jaw functional impairment in daily activities. Difficulties with chewing tough food and yawning were the most reported items. Although limited mouth opening has been reported by previous studies [16, 17, 23], voluntary mouth opening was less affected in the current study. One possible explanation is that yawning is a rapid, involuntary motor act and may provoke pain during condylar translation [44].

The mean (SD) OHIP-14 score in this sample was 12.3 ± 8.2, compared with only 10% of individuals in the Swedish population scoring ≥ 11 [45]. Since each OHIP-14 point corresponds to 53.2 impacts per month [31], this score represents approximately 654 impacts per month, indicating substantial impairment in oral health-related quality of life. To our knowledge, only one study in patients with PsA (*n* = 33) has reported a mean (SD) OHIP-14 score of 7.7 ± 8.5 (German version with comparable items but a different subdomain structure) [46]. This difference may be explained by the inclusion of participants with both PsA and TMD in our study. The most affected subdomains were orofacial pain (3.3 ± 2.4; e.g., aching in the mouth and discomfort when eating) and orofacial appearance (2.7 ± 1.8; self-consciousness and feeling tense). Part of these findings may be explained by TMD symptoms; however, dental status which may influence jaw function and oral health-related quality of life [43], was not assessed. PsA-related inflammation has been linked to osteoporosis [47] which has also been associated with reduced jawbone density and tooth loss. Although 40.7% of women in this study reported poor oral health, this was not clinically assessed. The orofacial appearance domain likely captures self-consciousness and emotional distress related to perceived oral appearance, consistent with the known psychological burden in rheumatic diseases [46].

The present findings suggest a complex relationship between PsA and TMD. Although 56% of participants perceived dental staff as knowledgeable about their disease, the limited reported beneficial effect of counselling may reflect challenges in managing this complex condition.

### Strengths and limitations

To our knowledge, this is the first epidemiological study in women with PsA to specifically stratify TMD-related findings by psoriasis history. A key strength is the comprehensive assessment of TMD within a biopsychosocial framework, incorporating a broad range of sociodemographic, disease-related, health-related, and patient-reported outcome measures, including validated instruments. However, the cross-sectional design limits causal inference. The low participation rate and recruitment from a patient association may have resulted in selection bias. Given the self-reported nature of the survey and the lack of clinical examination, participants might have provided inaccurate or incomplete information for some of the variables, leading to recall and reporting bias. PsA and psoriasis had been diagnosed within the healthcare system; however, these diagnoses were reported by the participants and were not independently verified from medical records. Therefore, some misclassification of PsA or psoriasis history is possible. The inclusion of three participants with additional inflammatory rheumatic diagnoses may also affect the internal validity of the study. Although these participants met the study inclusion criteria, the possibility of additional unreported diagnoses in the sample cannot be excluded. The small sample size, including only women, restricts generalizability to men and may also affect representativeness. Nevertheless, the impact of these biases on the estimated figures cannot be assessed.

## Conclusion

Women with PsA reported TMD symptoms and impaired patient-reported outcomes, with higher proportions reported in the psoriasis-history group. Self-reported beneficial effects of systemic and TMD-related treatments varied between groups. These exploratory findings warrant further investigation in larger studies. The complex relationship between PsA and TMD highlights the need for collaboration between dental and healthcare professionals. Further studies with larger samples are recommended.

## Supplementary Information

Below is the link to the electronic supplementary material.

Supplementary Material 1

Supplementary Material 2

## Data Availability

The data are not publicly available due to their sensitive nature, including information on health. For more information, please contact the corresponding author NY.
