# Supplementary material for: Temporomandibular disorders in women with psoriatic arthritis by history of psoriasis: a cross-sectional study
Source: Rheumatol Int. 2026 Oct 14;46(11):286. doi: 10.1007/s00296-026-06318-7 (PMC13668928; doi:10.1007/s00296-026-06318-7)
Supplement: Supplementary file 2 [file 296_2026_6318_MOESM2_ESM.docx]

**Supplementary Information**

**Table 1.** Classification of medication with self-reported beneficial effect for PsA after 3–6 months of use in the sample (n = 27).

| **Medication Classification** | **Representative examples** |
| --- | --- |
| csDMARDs | Methotrexate, leflunomide, sulfasalazine, hydroxychloroquine |
| bDMARDs | TNF inhibitors (etanercept, adalimumab, infliximab); IL-17 inhibitor (secukinumab); T-cell costimulation inhibitor (abatacept); IL-12/23 inhibitor (ustekinumab) |
| tsDMARDs | JAK inhibitor (baricitinib) |
| NSAIDs | Diclofenac, ketoprofen, naproxen, ibuprofen, celecoxib, etoricoxib |
| Analgesics | Paracetamol, tramadol, orphenadrine, buprenorphine, codeine |

**DMARDs,** disease-modifying antirheumatic drugs; **csDMARDs**, conventional synthetic disease-modifying antirheumatic drugs; **bDMARDs**, biologic disease-modifying antirheumatic drugs; **tsDMARDs**, targeted synthetic disease-modifying antirheumatic drugs; **NSAIDs**, non-steroidal anti-inflammatory drugs

**Table 2.** JFLS-8 items and total scores in women with PsA and TMD symptoms. Values are shown as median (first quartile–third quartile).

| **JFLS-8 items Classification** | **With**  **psoriasis history**  **(n=10)** | **Without**  **psoriasis history**  **(n=16)**  **(n=16)** | **Total**  **(n=26)** |
| --- | --- | --- | --- |
| **JFLS-1** Chew tough food | 4 (2-6) | 4.5 (3-7) | 4 (3-6) |
| **JFLS-2** Chew chicken | 1.5 (0–3) | 1 (0-3) | 1 (0-3) |
| **JFLS-3** Eat soft food requiring no chewing | 0 (0–1) | 0 (0-0) | 0 (0-0) |
| **JFLS-4** Open wide enough to drink from a cup | 0 (0-3) | 0 (0-0.5) | 0 (0-1) |
| **JFLS-5** Swallow | 0.5 (0-3) | 0 (0-3) | 0 (0-3) |
| **JFLS-6** Yawn | 2.5 (1-4) | 3 (0-5.5) | 3 (0-5) |
| **JFLS-7** Talk | 0.5 (0-3) | 0 (0-1) | 0 (0-1) |
| **JFLS-8** Smile | 0 (0-3) | 0 (0-0.5) | 0 (0-1) |
| **Total score** | 18 (5 – 22) | 11.5 (8 –17.5) | 13.5 (7 –21) |

**Table 3.** OHIP-14 items, sum scores and dimensions in women with PsA and TMD symptoms. Values are shown as the mean value ± standard deviation [median; [first quartile–third quartile].

| **OHIP-14 Item** | **With**  **psoriasis history**  **(n=10)** | **Without**  **psoriasis history**  **(n=16)** | **Total sample**  **(n=26)** |
| --- | --- | --- | --- |
| **1.** Trouble pronouncing | 0.5 ± 0.7 [0; 0–1] | 0.2 ± 0.5 [0; 0–0] | 0.3 ± 0.6 [0; 0–0] |
| **2.** Taste worsened | 0.5 ± 0.7 [0; 0–1] | 0.2 ± 0.7 [0; 0–0] | 0.3 ± 0.7 [0; 0–0] |
| **3.** Painful aching in the mouth | 2 ± 1.3 [2; 1–3] | 1.8 ± 1.3 [2; 1–2] | 1.9 ± 1.3 [2; 1–3] |
| **4.** Uncomfortable eating | 1.9 ± 1.4 [1.5; 1–3] | 1.1 ± 1.4 [1; 0–2] | 1.4 ± 1.4 [1; 0–2] |
| **5.** Been self-conscious | 0.7 ± 1.1 [0; 0–1] | 0.7 ± 1.2 [0; 0–1.5] | 0.7 ± 1.1 [0; 0–1] |
| **6.** Feeling of tension | 2.3 ± 1.4 [2.5; 1–3] | 1.8 ± 1.2 [2; 1–2] | 2.0 ± 1.3 [2; 1–3] |
| **7.** Diet unsatisfactory | 0.8 ± 0.8 [1; 0–1] | 0.5 ± 0.7 [0; 0–1] | 0.6 ± 0.8 [0; 0–1] |
| **8.** Interrupting meals | 0.9 ± 1.0 [0.5; 0–2] | 0.3 ± 1.0 [0; 0–0] | 0.6 ± 1.0 [0; 0–1] |
| **9.** Difficult to relax | 1.9 ± 0.9 [2; 1–2] | 1.4 ± 1.4 [1; 0–2] | 1.6 ± 1.2 [2; 1–2] |
| **10.** Embarrassed | 0.4 ± 0.7 [1; 0–1.5] | 0.4 ± 0.8 [0; 0–0.5] | 0.4 ± 0.8 [0; 0–1] |
| 1**1.** A bit irritable with other people | 1.1 ± 1.2 [1; 0–1] | 0.5 ± 0.8 [0; 0–1] | 0.7 ± 1.0 [0; 0–1] |
| **12.** Difficulty doing usual jobs | 1.7 ± 1.4 [2; 0–3] | 0.2 ± 0.5 [0; 0–0] | 0.8 ± 1.2 [0; 0–2] |
| **13.** Feeling of life in general was less satisfying | 1.4± 0.7 [1; 1–2] | 0.4 ± 0.6 [0; 0–1] | 0.8 ± 0.8 [1; 0–1] |
| **14.** Totally unable to function | 0.6± 0.8 [0; 0–1] | 0.1 ± 0.3 [0; 0–0] | 0.3 ± 0.6 [0; 0–0] |
| **OHIP-14 sum score** | 16.7± 7.7 [16; 10–21] | 9.6± 7.4 [9; 3.5–  15.5] | 12.3 ± 8.2 [10.5; 7–18] |
| **Oral function** | 1± 1.3 [0.5; 0–2] | 0.4± 1.0 [0; 0–0] | 0.7± 1.1 [0; 0–1] |
| **Orofacial pain** | 3.9± 2.5 [3.5; 2–7] | 2.9± 2.4 [2.5; 1.5–4] | 3.3± 2.4 [3; 2–7] |
| **Orofacial appearance** | 3± 1.9 [3; 2–4] | 2.5± 1.9 [2; 1.5–4] | 2.7± 1.8 [2; 2–4] |
| **Psychosocial impact** | 2.0± 1.2 [1.5; 1–3] | 0.4± 0.8 [0; 0–1] | 1.1± 1.2[1; 0–1] |
